# Industrial-Scale Cleaning Solutions for the Reduction of *Fusarium* Toxins in Maize

**DOI:** 10.3390/toxins14110728

**Published:** 2022-10-25

**Authors:** Michelangelo Pascale, Antonio F. Logrieco, Vincenzo Lippolis, Annalisa De Girolamo, Salvatore Cervellieri, Veronica M. T. Lattanzio, Biancamaria Ciasca, Anna Vega, Mareike Reichel, Matthias Graeber, Katarina Slettengren

**Affiliations:** 1 Institute of Food Sciences (ISA), National Research Council of Italy (CNR), 83100 Avellino, Italy; 2 Institute of Sciences of Food Production (ISPA), National Research Council of Italy (CNR), 70126 Bari, Italy; 3 Bühler AG, 9240 Uzwil, Switzerland; 4 Eurofins WEJ Contaminants, 21079 Hamburg, Germany

**Keywords:** maize, deoxynivalenol, zearalenone, fumonisins, remediation, mechanical cleaning, optical sorting

## Abstract

Grain cleaning is the most effective non-destructive post-harvest mitigation strategy to reduce high levels of mycotoxins on account of the removal of mold-infected grains and grain fractions with high mycotoxin content. In this study, the reduction in the concentration of some co-occurring *Fusarium* toxins in maize, namely deoxynivalenol (DON), zearalenone (ZEA) and fumonisins B1 and B2 (FBs), was evaluated at an industrial-scale level by mechanical removal (sieving and density separation) of dust, coarse, small, broken, shriveled and low-density kernels and/or optical sorting of defected kernels. Samples were dynamically collected according to the Commission Regulation No. 401/2006 along the entire process line. Mycotoxin analyses of water–slurry aggregate samples were performed by validated LC methods. Depending on the contamination levels in raw incoming maize, the overall reduction rates ranged from 36 to 67% for DON, from 67 to 87% for ZEA and from 27 to 67% for FBs. High levels of DON, ZEA and FBs were found in all rejected fractions with values, respectively, up to 3030%, 1510% and 2680%, compared to their content in uncleaned maize. Results showed that grain cleaning equipment based on mechanical and or optical sorting technologies can provide a significant reduction in *Fusarium* toxin contamination in maize.

## 1. Introduction

Pathogenic fungi of the genus *Fusarium* are widespread in cereal-growing areas worldwide causing severe crop yield losses with consequent economic losses. In addition, several *Fusarium* species colonizing cereals, under favorable environmental conditions, can produce and accumulate mycotoxins in grains, of which some are of notable concern for human and animal health [1,2,3,4].

Deoxynivalenol (DON), zearalenone (ZEA) and fumonisins B1 and B2 (FBs) are well-known *Fusarium* toxins associated to cereals, including maize. Their toxic effects on humans and animals have been studied for many years and several scientific opinions and evaluations on risks related to exposure estimates have been carried out by several authorities [5,6,7,8,9,10]. The International Agency for Research on Cancer (IARC) has classified fumonisin B1 (FB1) as possibly carcinogenic to humans (Group 2B), whilst DON and ZEA are not classifiable as to their carcinogenicity to humans (Group 3) [11].

In order to protect human and animal health, maximum limits for *Fusarium* toxins (mainly DON, ZEA and FBs) in cereals and cereal-based products have been established/recommended in several countries worldwide, including the European Union [12,13,14,15].

Current recommended practices for the prevention and reduction in *Fusarium* toxin contamination in cereals are based on Good Agricultural Practices (GAP) and Good Manufacturing Practices (GMP) [16,17,18]. However, in some agricultural seasons the levels of *Fusarium* toxins in harvested crops could be higher than the maximum permitted levels due to climatic conditions favorable to the growth of toxigenic fungi, with consequent mycotoxin accumulation in grains. The contaminated batches, depending on the levels of contamination, should be destroyed or used as animal feed or as biomass for biofuel production with consequent economic losses for the farmers. To avoid this, several post-harvest decontamination strategies using physical, chemical or biological approaches have been investigated with the aim of reducing mycotoxin contamination in grains [19,20,21,22,23,24,25,26,27]. In particular, physical methods (i.e., sieving, aspiration, gravity separation, manual or optical sorting) removing visibly moldy, low-density, infected, colored/discolored, broken and/or damaged kernels, as well as fine materials and dust, have shown to be effective in reducing mycotoxins in cereals and other commodities [28,29,30,31,32,33,34,35].

Several studies on the fate of *Fusarium* mycotoxins during the processing of wheat have been carried out at laboratory or pilot level showing that cleaning and sorting steps are effective solution in removing toxins contaminated fractions. However, the effects of mycotoxins reduction significantly varied depending on the level of contamination and the amount of rejected fractions during the processing [36,37,38,39]. A recent study has showed that the removal of small kernels by a laboratory sieve also reduces the content of *Fusarium* mycotoxins in oats thus improving the grain quality [40].

Regarding maize, a comprehensive review on the fate of mycotoxins, including the *Fusarium* toxins DON, ZEA and FBs, during the primary food processing of maize has been recently published by Schaarschmidt and Fauhl-Hassek [41]. Changes in *Fusarium* toxins DON, ZEA and FBs level during cleaning of maize largely varied depending on batches and type of mycotoxins, as well as processing procedures. As an example, a reduction in FBs concentrations between 30–90% in small batches of maize was obtained by manual sorting [42,43,44] whereas cleaning based on sieving reduced FBs concentrations by 25–70% [45,46,47]. Analogously to manual sorting, optical sorting has been demonstrated to efficiently reduce the levels of fumonisins (and aflatoxins) in maize [48,49].

Dry milling has shown to significantly reduce DON and ZEA concentration in grits and flour, as well as other minor *Fusarium* toxins including 3-acetyl-DON (3-ADON), 15-acetyl-DON (15-ADON), nivalenol (NIV), T-2 and HT-2 toxin and moniliformin (MON) in maize [50,51].

Industrial processing may not always reflect what is observed in laboratory and pilot-scale experiments; however, the few studies carried out at industrial level have shown the efficacy of cleaning and sorting in reducing the level of mycotoxins in cereal grains. In particular, the efficacy of maize cleaning steps on aflatoxin B1 (AFB1) and FB1 contamination levels has been evaluated in an industrial scale process aimed to assess the distribution of these mycotoxins in fractions derived from the dry-milling of two maize lots contaminated at different levels. The cleaning step reduced AFB1 and FB1 levels by 8–57% and 11–34%, respectively [52]. In a similar study aimed to evaluate the distribution of FBs in maize dry-milling products and by-products, grain-cleaning using a dry stoner, an intensive horizontal scourer, a vibrating aspirator and an optical sorter reduced FBs by about 42% [53]. More recently, a continuous cleaning line combining both mechanical and optical sorting technologies at industrial scale level has been shown to be an efficient solution for reducing aflatoxins (AFBs) in maize. Batches of biomass/feed quality maize contaminated by AFBs were converted into feed/food quality maize. Aflatoxin reductions from 65% to 84% with respect to the uncleaned products were observed [54]. Very high levels of AFBs (up to 490 µg/kg) were found in the rejected fractions, showing the effectiveness of removing small and broken kernels, dust/fine particles, defected kernels for reducing mycotoxin contamination in maize. Industrial cleaning processes involving scouring, aspiration, and optical sorting reduced mean MON content in maize by 47%. Similarly, a content mean reduction in 52% was achieved for FBs contamination [51].

At our knowledge, to date, no targeted study on the assessment of the effectiveness of cleaning/sorting technologies in industrial grain processing for the simultaneous reduction in legislated *Fusarium* toxins (i.e., DON, ZEA and FBs) has been carried out. The aim of this study was to evaluate the efficacy of industrial-scale dry cleaning equipment in reducing DON, ZEA and FBs in naturally contaminated maize. Two studies have been carried out at industrial level in Italy and Spain, respectively, for investigating the effect of cleaning solutions on the reduction in *Fusarium* mycotoxins, i.e., DON, ZEA and FBs in maize. The first study was carried out to evaluate the performances of a high-capacity optical sorting machine in removing contaminated kernels from naturally highly contaminated maize batches; the second study was aimed to evaluate the performance of a cleaning industrial line combining both mechanical (separator, aspirator, concentrator) and optical sorting in reducing the content of the above-mentioned *Fusarium* toxins in maize. The mass balance of the three mycotoxins after cleaning was carried out in order to verify the accuracy of the results.

## 2. Results

### 2.1. First Study

In the first study the total amount of reject fractions with coloured/discoloured and defective maize kernels of the three batches of maize naturally contaminated with DON, ZEA and FBs accounted to 5% (Table 1).

Mycotoxin levels in the three batches ranged from 3200 to 17,400 µg/kg for DON, from 660 to 4460 µg/g for ZEA and from 2520 to 6540 µg/kg for FBs with batch C being the least contaminated one for all mycotoxins (Table 2). The fraction rejected from the sorter contained higher levels of mycotoxins compared to the unprocessed maize (incoming fraction). In the case of DON and FBs, the increment of mycotoxin levels was quite similar in the three batches, accounting to approximately 800% for DON and 250% for FBs. In the case of ZEA a higher variability was observed within the three batches, and values ranged from 400 to 1400%. Consequently, low levels of mycotoxins were observed in the cleaned maize with a reduction between 44–67% of DON, 67–87% of ZEA and 27–28% of FBs, with respect to their content in incoming maize (Table 2).

### 2.2. Second Study

In the second study two batches (17 tons each) of maize naturally contaminated with DON, ZEA, FB1 and FB2 were processed by a cleaning industrial line comprising a separator coupled with an aspirator, a concentrator and an optical sorter (mass flow rate: 17 tons/h). Percentages of rejected fractions containing broken/damaged kernels, fine and foreign materials collected from separator, aspirator, concentrator and optical sorter ranged from 0.1 to 3% with separator providing the highest rejected amount (Table 1). The total amount of reject fractions accounted to 6.4% and 4.0% for batches A (maize from France) and B (maize from Spain), respectively. Mycotoxin levels determined in the sampled fractions of the two replicates of the batches A and B are reported in Table 3. Levels in the uncleaned maize were between 220–350 µg/kg for DON, 40–55 µg/kg for ZEA and 1705–1765 µg/kg for FBs. In all cases, these levels were far below the EU maximum levels established for these mycotoxins in uncleaned maize (i.e., 1750 µg/kg for DON, 350 µg/kg for ZEA and 4000 µg/kg for FBs [13]. After the different cleaning steps through TAS^TM^, concentrator MTCB^TM^ and SORTEX^®^ Z+, mycotoxins levels in the cleaned maize were 10 µg/kg for ZEA and between 105–200 µg/kg for DON and 580–1160 µg/kg for FBs, corresponding to a reduction in 75–82%, 36–52% and 34–67%, respectively. The reduction in DON and ZEA between the two batches of maize was similar, while in the case of FBs it was higher in the Spanish batch.

**Figure 1:**
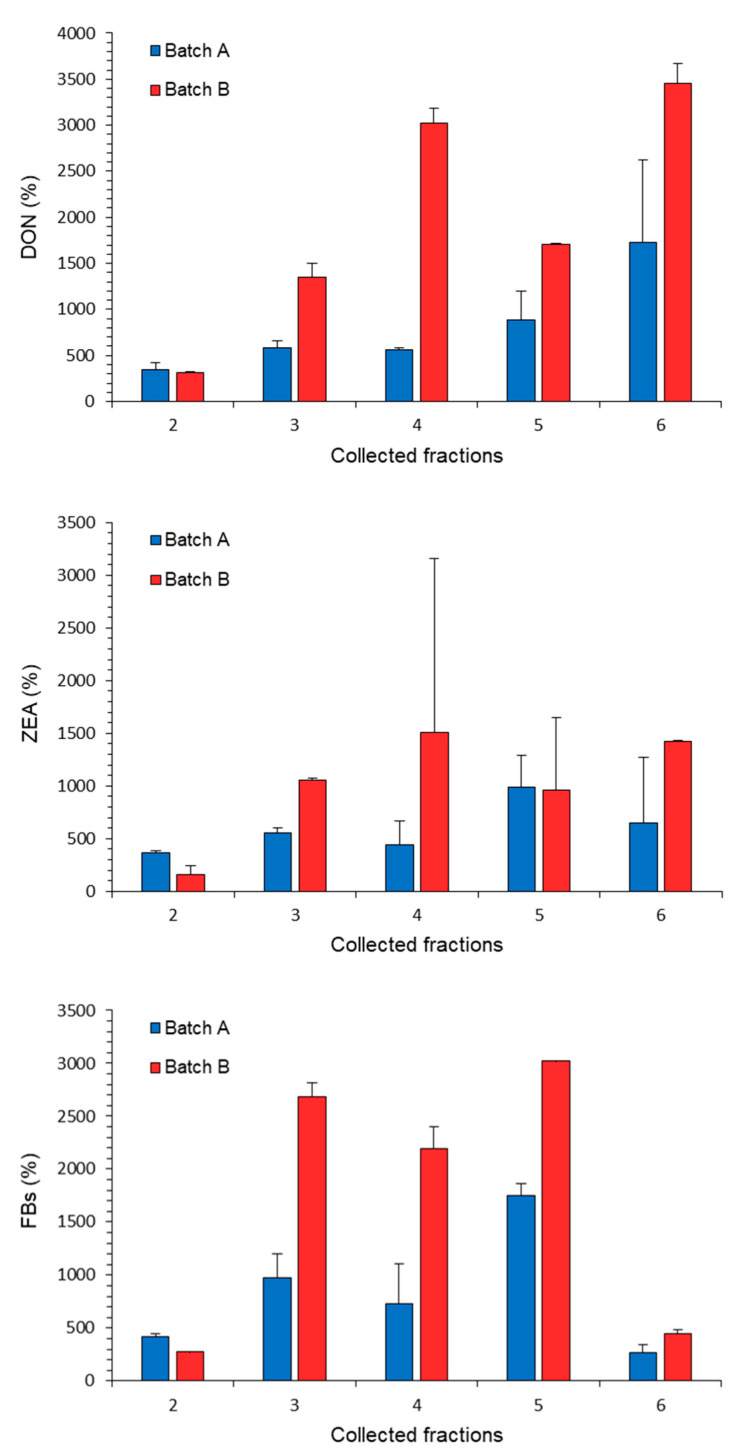
Content (%) of deoxynivalenol (DON), zearalenone (ZEA) and total fumonisins (FBs) in rejected fractions from separator (2, small, broken and fine material and 3, coarse and fine material), from aspirator (4, dust and husks particles), from concentrator (5, low density kernels), from optical sorter (6, coloured and discoloured and defective maize kernels) of batch A (French maize) and batch B (Spanish maize). Each bar corresponds to the average content of mycotoxin in each batch (two replicates) with respect to the relevant content in uncleaned maize + standard deviation (fraction 1, incoming material).

This reduction corresponded to an incremented concentration of mycotoxins in the rejected fractions (fractions 2–6) for all targeted mycotoxins. Specifically, the content (%) of mycotoxins in the fractions from batches A and B of maize was between 162–417% in fractions 2, 550–2683% in fractions 3, 444–2194% in fractions 4, 962–3026% in fractions 5 and between 267–3457% in fractions 6, with respect to the raw material (uncleaned maize) (Figure 1), with a more evident effect in maize from Spain (batch B).

### 2.3. Mass Balance

A mass balance calculation was applied to quantitatively estimate the distribution of DON, ZEA and FBs among fractions obtained during the industrial-scale maize cleaning as compared to raw material. Mass balance results for study 1 (batches A, B and C) and study 2 (batches A1, A2, B1 and B2) are reported in Table 4. Overall, the mass balance in the study 1 was between 80–108% for the three mycotoxins. Similar results were also obtained in the study 2 for DON (79–105%) and FBs (88–114%), while lower and slightly more variable results were obtained in the case of ZEA (37–62%). These latest results were probably related to the low contamination levels of ZEA in the starting maize for study 2 (40–55 µg/kg) that were close to the limit of quantification of the method (35 µg/kg), with respect to those in study 1 (661–4460 µg/kg). The low levels of ZEA contamination in batches A1, A2, B1 and B2 led to a higher analytical error. By excluding ZEA results in study 2, the overall results indicated a reliable sampling plan, a good accuracy of analytical data and a suitability of the industrial-scale cleaning studies described in the present paper.

## 3. Discussion

Cleaning of cereals allows the removal of foreign materials and broken, shrivelled, damaged and low-density kernels. The process is commonly used before storage and/or milling. This physical procedure has been shown to be effective in reducing mycotoxin contents by removing highly contaminated material at the early stage of the food and feed chain and to prevent fungal colonization during storage.

Traditional cleaning techniques include the manual sorting-out of small, broken, and low-density grains. Although it can be quite effective in reducing *Fusarium* toxins, the automated optical sorting represents a more specific strategy for removing *Fusarium*-infected grains and related toxins, even though they require more cost-intensive equipment [26,36,41]. Results reported in the present paper confirm that optical sorting represents an effective strategy for reducing mycotoxins along the entire chain of industrial maize processing. Furthermore, the integrated process solutions removing mechanically *Fusarium*-infected maize kernels, as well as maize fractions based on their characteristics (i.e., specific gravity and optical properties), makes this cleaning procedure suitable for managing *Fusarium* mycotoxin in maize. However, in general, most of the literature data describe the combined effect of sorting, cleaning and milling on the reduction in mycotoxins in wheat and maize. The majority of data are for DON and FBs, and at lesser extent for other *Fusarium* toxins such as T2/HT2, 3-ADON, 15-ADON and NIV, while limited information is available for ZEA [24,26,36,39,41,55]. In a study undertaken to examine the efficiency of a high-speed optical sorting of wheat kernels, an average reduction in DON contamination levels of 51%, with respect to the concentration in unsorted wheat was observed. Successive cleaning steps were successful at further reducing the concentration of DON [56]. In another work, the removal of *Fusarium*-contaminated grains from wheat using optical sorters successfully reduced the DON and NIV concentrations in the cleaned wheat by approximately 50% [57]. Another application of optical sorting was reported by Carmack et al. [33] for the selection of breeding lines of wheat with enhanced *Fusarium* head blight resistance, i.e., with lower levels of DON and *Fusarium*-damaged kernels. Results obtained herein on the effect of sorting and cleaning on DON removal from maize (36–67%) are in line with the range reported in the literature, while a similar comparison is not reliable for ZEA because of limited data availability on it. A significant reduction in DON levels was also observed after the application of colour sorting to 20 different samples of wheat, with an average level of approximately 12% [34].

In a study evaluating the redistribution of 16 *Fusarium* toxins, including DON and ZEA, dry milling of two batches of maize at industrial level, a marked increment of mycotoxin levels (396–807% for DON and 1400–1743% for ZEA) was found in screenings (containing small and broken and, therefore, heavily mouldy and toxin contaminated kernels) compared to the mycotoxin content in the starting maize [50].

Concerning FBs, in a study carried out using a dual wavelength high-speed commercial sorter on white maize contaminated at different levels, a reduction ranging from 29 to 96% of FBs was observed by rejecting from 4 to 9% of maize [49]. In another work, Westhuizen et al. [44] described the application of hand-sorting of home-grown maize kernels under laboratory-controlled conditions by reporting a removal of FBs by 71% with an additional 13% after a 10-min ambient temperature water wash. During the dry-milling process of four lots of maize, the cleaning operation, mainly carried out using an optical sorter, reduced the level of FBs in the cleaned maize from 43% to 76% depending on the specific set up of the optical sorter used for each lot of maize which was milled in different growing seasons [51]. A low-cost sorter prototype (the ‘DropSort’ device) separating maize based on kernel bulk density was effective in reducing FBs in maize. In a further study, the DropSorter was combined with size sorting to separate grain samples into large + heavy kernels and small + light kernels and FBs reduction was up to 98% [32].

Data on the effects of industrial-scale optical sorting on simultaneous reduction in *Fusarium* toxins in cereals are very limited, and only few describe their application to FBs. Vanara et al. [53] reported a 42% reduction in FBs after cleaning of maize during the milling process using a dry stoner, an intensive horizontal scourer, a vibrating aspirator and an optical sorter. Similarly, two different batches of maize obtained after cleaning steps including a separator with aspirator, a dry de-stoner and an intensive scourer coupled with an aspirator, achieved to lower the FB1 level by 11% and 34%, respectively, compared to the uncleaned maize [52]. In a study aimed to evaluate the fate of AFBs and FBs during the processing of maize for the production of makume and owo, maize-based foods common in Benin (West Africa), the sorting and winnowing of different batches of maize reduced FBs by 69% and 44%, respectively [42]. A certain agreement of results for FBs removal after sorting was observed among results reported in the present study (27–67%, Table 3 and Table 4) and the literature cited herein (11–98%) [32,42,44,49,51,52,53]. On the other hand, Generotti et al. [58], reported that the maize cleaning step through a system characterized by a separator with aspirator and sieve, a magnet and an optical sorter did not cause a reduction in FBs content in cleaned maize.

The effect of cleaning in terms of mycotoxin reduction can greatly vary depending on the levels of contamination in the raw material and from the quality of a batch, which can be affected by the cultivar, weather conditions and cultivation practices [26,36,41]. Although in the present study the effect of contamination levels cannot be evaluated on account of the comparable mycotoxins content in the batches used for study 2, the higher percentage of total rejected fractions observed in French maize with respect to Spanish maize (Table 1) can be justified by their different origin having potential differences in terms of weather conditions and cultivation practices, cultivars and harvest techniques. The high variability in mycotoxin distribution among fractions after cleaning resulted from the sampling plan, as well as from the amount of rejected fractions. The effects of *Fusarium* toxins reduction were more evident in Spanish maize (batch B), with respect to French maize (batch A) (Figure 1). A possible explanation of these results could be related to the different total amount of discarded fractions during the overall cleaning process between the two batches, i.e., 6.4% for French maize and 4% for the Spanish one, also because the mycotoxin levels in the two batches of uncleaned maize materials were quite similar. Moreover, a higher increment of concentration in the fraction 6 rejected from the sorter was observed for DON as compared to that of study 1. Maybe this behaviour was attributable to the lower DON level in the uncleaned maize of study 2 (less than 400 µg/kg) compared to that in the uncleaned maize of study 1 (3200–17,400 µg/kg, depending on the batch). On the other hand, ZEA results in the sorter rejected fraction confirmed those reported for study 1, either in terms of high variability than in terms of increase in concentration (up to approximately 1400%). Similarly, results for FBs increase in the rejected fractions were in the same range as those observed for study 1. However, the rejected fraction from the sorter (fraction 6) showed a lower increase in FBs concentration compared to the other rejected fractions (i.e., from 2 to 5).

## 4. Conclusions

The present study evaluates the effect of industrial-scale cleaning equipment on the simultaneous reduction in DON, ZEA and FBs in uncleaned maize. Specifically, two studies were carried out; in the first study maize samples were cleaned by an optical sorter which removed foreign bodies and kernels with visual defects; in the second one, maize samples were mechanically cleaned with a separator, an aspirator and a density separation machine and then optically sorted. Starting materials (uncleaned maize), final materials (cleaned maize) and rejected fractions were analysed for mycotoxins content. A reduction in mycotoxins was observed in both studies. In particular, the first study clearly showed the effectiveness of the use of optical sorting at industrial level in simultaneously reducing DON, ZEA and FBs in maize. Similarly, the second study showed that the combination of mechanical cleaning and optical sorting were able to reduce mycotoxins level in maize. In both cases, mycotoxins were accumulated in the rejected fractions. The calculated mass balance confirmed the reliability and accuracy of the tested approaches.

Results reported herein confirm that cleaning procedures based on mechanical and/or optical sorting technologies are effective in the reduction in DON, ZEA and FBs in uncleaned maize. The industrial scale of these experiments makes the obtained results very reliable by indicating that the application of cleaning procedures contributes to fulfil food safety requirements and could be part of a successful strategy for managing *Fusarium* mycotoxins in maize.

## 5. Materials and Methods

### 5.1. Materials and Reagents

Analytical-grade solvents, *o*-phthaldialdehyde (OPA), 2-mercaptoethanol, sodium tetraborate and phosphate buffered saline (PBS) tablet, were purchased either from Mallinckrodt Baker (Milan, Italy) or Sigma (St. Louis, MO, USA). Ultrapure water was produced by a Millipore Milli-Q system (Millipore, Bedford, MA, USA). Standards of DON, ZEA, FB1 and FB2 were purchased from Sigma. DONTest, ZearalaTest and FumoniTest^TM^ Wide Bore immunoaffinity columns were purchased from Vicam L.P. (A Waters Business, Milford, MA, USA).

### 5.2. Samples and Cleaning Processes

In the first study three batches of maize naturally contaminated with DON, ZEA, FB1 and FB2 (25 tons each), namely A, B and C, slightly pre-cleaned by mechanical sorting were cleaned by an optical sorter (SORTEX A5 BRBX, Buhler AG, Uzwil, Switzerland). Sortex A5 consists of five modules, each of a width of 300 mm, with the machine capable of processing a total of 10–20 tons per hour. Each module of the sorter has visible and shortwave Infrared (InGaAs) cameras both front and rear. The colour cameras were setup to detect colour and spot defects from the maize. The IR cameras were setup to remove the challenging foreign material not targetable with the visible system. Once an object had been identified as defective the decisions were sent to a bank of valves and a targeted compressed air pulse was used to remove the object from the product stream, thus ensuring maximum food safety of the cleaned product. The study was carried out for research purposes in North Italy in 2015 in a plant able to process 25 tons of raw maize per hour. The scheme of the industrial sorting line is shown in Figure 1.

**Figure 2:**
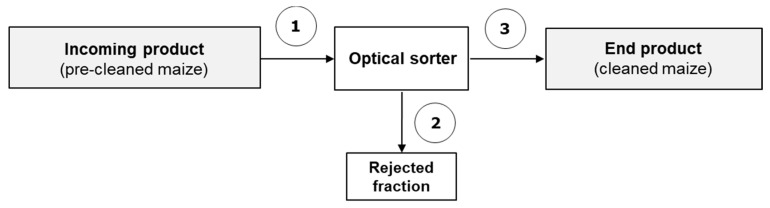
Scheme of the industrial cleaning line and sampling points (numbered) for study 1. Fractions: 1, slightly pre-cleaned maize; 2, coloured/discoloured and defective maize kernels from sorter; 3, cleaned maize. Fractions 1, 2, 3: dynamic sampling.

The second study was carried out in Spain in 2018 in a plant able to process 17 tons/h of raw maize. The scheme of the industrial cleaning line is shown in Figure 2.

In study 2, two batches of maize (17 tons each), namely A (from France) and B (from Spain), naturally contaminated with DON, ZEA, FB1 and FB2 were mechanically cleaned and optically sorted using industrial-scale cleaning equipment. The equipment included a sieving machine (TAS^TM^), consisting of a separator and an aspirator, a density separation machine, consisting of a concentrator (MTCB^TM^), as well as an optical sorting machine (SORTEX^®^ Z+). The sieving separator removed small/broken/fine materials (fraction 2) and coarse/fine (fraction 3) kernels, the aspirator removed dust and husk particles (fraction 4), while the concentrator classified maize fractions into high-, mixed- and low-density materials and eliminated lighter maize fractions (fraction 5). The sorter was equipped with an enhanced InGaAs camera and climate control and removed kernels with visual signs of contamination (fraction 6). The outlet of the sorter was the cleaned maize, representing the end product (fraction 7).

### 5.3. Sampling

Samples were taken dynamically according to the Commission Regulation No. 401/2006 along the entire process line, including cleaned and rejected project streams [59].

For study 1 incremental samples ranging from 10 to 100 (100–300 g each) were collected from the sorter at regular intervals according to the sampling protocol. For study 2 incremental samples ranging from 5 to 60 (100–300 g each) were collected for each batch of maize at sampling points 1–7 from opening slits of the plants at regular intervals. Two replicates per batch, for a total of four studies, were carried out, i.e., A1, A2, B1 and B2.

**Figure 3:**
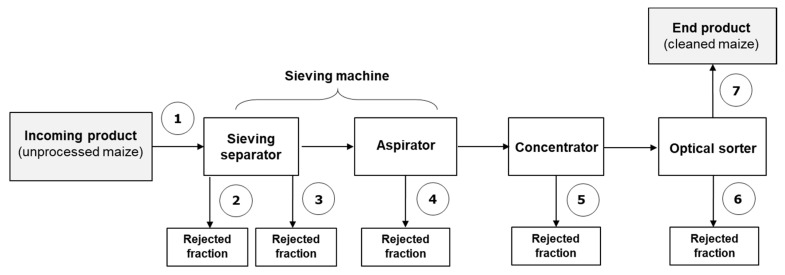
Scheme of the industrial cleaning line and sampling points (numbered) for study 2. Fractions: 1, unprocessed maize: 2, small, broken and fine material from separator; 3, coarse and fine material from separator; 4, dust and husks particles from aspirator; 5, lighter maize fractions from concentrator; 6, coloured/discoloured and defective maize kernels from sorter; 7, cleaned maize. All fractions were dynamically sampled.

For both studies, the number of incremental samples and the weight of the aggregate samples submitted to analysis are reported in Table 5. Sampling points are indicated in Figure 2 and Figure 3. Samples were maintained at +4 °C until the mycotoxin analysis was performed.

### 5.4. Mycotoxins Analysis

To minimize subsampling errors aggregate samples of fractions weights higher than 5 kg were slurry-mixed with water in matrix:water ratio 1:1 (*w*:*w*) for 10 min using the Silverson EX high share mixer (Silverson Machines Ltd., Waterside, Chesham, UK) as described by Pascale et al. [54]. For aggregate samples of 1–2 kg, an Ultra Turrax IKA T25 (IKA Werke GmbH & Co. KG., Staufen, Germany) was used for preparation of slurries and for recovery experiments. Unprocessed maize samples (study 2) were preliminarily analysed by LC-MS/MS [60] to screen the simultaneous occurrence of mycotoxins. Low levels of AFBs (up to 0.25 µg/kg), ochratoxin A (up to 0.15 µg/kg), T2 toxin (up to 10 µg/kg), HT2 toxin (up to 15 µg/kg) and beauvericin (up to 5 µg/kg) were observed. Then, the analysis of DON, ZEA, FB1 and FB2 in the water–slurry samples was carried out at CNR using validated HPLC methods.

#### 5.4.1. Analysis of DON

Analysis of DON was performed according to [61] for the determination of DON in cereals and cereal products with some modifications. Briefly, aliquots of slurry (50 g) were extracted with 75 mL PBS by blending at high speed for 2 min (Sorvall Omnimixer). The extracts were filtered through Whatman No. 4 filter paper (Whatman, Maidstone, UK) followed by glass microfiber filter Whatman GF/A (Whatman). One ml of filtered extract was cleaned up through DONTest immunoaffinity column (VICAM) at a rate of about 1 drop/second. The column was washed with 2 × 5 mL water at a flow rate of 1–2 drops/s and DON was eluted with 2 × 0.75 mL methanol in a 4-mL vial. The eluted extract was gently dried under a nitrogen stream at about 50 °C and reconstituted with 250 μL of LC mobile phase (water:methanol, 85:15, *v/v*). An aliquot of 10 µL of reconstituted extract (equivalent to 0.01 g sample matrix) was injected into the chromatographic apparatus by full loop injection. The LC system consisted of the ultra-high performance liquid chromatography instrument (UHPLC) Agilent 1290 Infinity (Agilent Technologies, Santa Clara, CA, USA) equipped with a pump, degasser, column oven, auto sample injector and a PDA detector. The chromatographic separation of DON was obtained using a Zorbax Eclipse XDB-C18 column (150 mm × 2.1 mm, 1.8 µm) and an isocratic mobile phase of water:methanol (85:15, *v/v*) at a flow rate of 0.4 mL/min. With these conditions, deoxynivalenol eluted within 9 min. Limit of detection (LOD), based on a signal to noise ratio of 3:1, was 10 µg/kg DON. Limit of quantification (LOQ), based on a signal to noise ratio of 10:1, was 35 µg/kg DON.

#### 5.4.2. Analysis of ZEA

Analysis of ZEA was performed according to [62] for the determination of ZEA in barley, maize and wheat flour, polenta, and maize-based baby food with some modifications. Briefly, aliquots of slurry (40 g) were extracted with 180 mL acetonitrile by blending at high speed for 2 min (Sorvall Omnimixer). The extracts were filtered through Whatman No. 4 filter paper (Whatman) and 10 mL of filtered extract was diluted with 90 mL PBS and filtered through Whatman GF/A (Whatman). Then, 20 mL of filtered extract was cleaned up through ZearalaTest immunoaffinity column (VICAM) at a rate of about 1 drop/second. The column was washed with 2 × 5 mL water at a flow rate of 1–2 drops/s and mycotoxins were eluted with 2 × 0.75 mL methanol in a 4-mL vial. After drying under a nitrogen stream at about 50 °C, the extract was reconstituted with 250 μL of LC mobile phase (water:acetonitrile:methanol, 46:46:8, *v/v/v*). An aliquot of 100 µL of reconstituted extract (equivalent to 0.08 g sample matrix) was injected into the chromatographic apparatus by full loop injection. The LC system consisted of a high-performance liquid chromatography instrument (HPLC) Agilent 1100 Series (Agilent Technologies) equipped with a pump, degasser, column oven, auto sample injector and a fluorescent detector (λ_ex_ = 274 nm; λ_em_ = 440 nm). The chromatographic separation of ZEA was obtained using a Symmetry C18 column (150 mm × 4.6 mm, 5 µm) (Waters) and an isocratic mobile phase (water:acetonitrile:methanol, 46:46:8, *v/v/v*) at a flow rate of 1.0 mL/min. With these conditions, ZEA eluted within 7 min. The LOD and LOQ values of the method were 10 µg/kg and 35 µg/kg ZEA, respectively.

#### 5.4.3. Analysis of FB1 and FB2

Extraction of FBs from maize was carried out according to [63] with some modifications. Briefly, aliquots of slurry (40 g) were extracted with a mixture (40 mL) of methanol:acetonitrile:water (31.25:31.25:37.50, *v/v/v*) by shaking for 20 min. After filtration through Whatman No. 4 filter paper (Whatman) the remaining solid material was extracted again with the extraction solvent (40 mL) by shaking for 20 min and the extract was filtered through the same filter paper. The two extracts were combined, and an aliquot of filtrate (10 mL) was diluted with PBS (40 mL) and filtered through Whatman GF/A (Whatman). Then a volume of filtered extract (10 mL) was cleaned up through FumoniTest^WB^ immunoaffinity column (VICAM). After elution, the column was washed with 10 mL PBS and FBs were eluted with 2 × 1 mL methanol followed by 2 × 1 mL water in a 4-mL vial. Then the extract was dried under a nitrogen stream at about 50 °C and reconstituted with 500 μL of water:acetonitrile (70:30, *v/v*). Sample extracts were derivatised with OPA reagent and analysed by HPLC according to the procedure described by De Girolamo et al. [64]. With these conditions, retention times of FB1 and FB2 were about 17 and 24 min, respectively. The LOD values were 70 µg/kg for FB1 and 40 µg/kg for FB2, while LOQ values were 240 µg/kg for FB1 and 140 µg/kg for FB2.

### 5.5. Mass Balance

For each mycotoxin and for each study, the mass balance (in %) was calculated by taking into account the amount of mycotoxin (mg) in the rejected fractions and in the final cleaned maize, with respect to the amount of mycotoxin (mg) in the incoming product (unprocessed maize) according to the formula [1].

(1) $$\mathrm{Mass}\;\mathrm{balance}\;(\%)=\sum \left[\mathrm{mycotoxin}\;\mathrm{amount}\;\mathrm{in}\;\mathrm{all}\;\mathrm{collected}\;\mathrm{fractions}\right]/\left[\mathrm{mycotoxin}\;\mathrm{amount}\;\mathrm{in}\;\mathrm{starting}\;\mathrm{maize}\;\left(\mathrm{unprocessed}\;\mathrm{maize}\right)\right]\;\times \;100$$

The information about the mass loss (%) was provided taking in account the technical specifications of the plants and from previous measurements carried out during the processing, before starting with the trials.

## Figures and Tables

**Table 1:** Average yields (%) of maize-cleaning sampled fractions.

Maize-Cleaning Fraction	Study 1		Study 2		
	Sampled Fraction	Yield (%) Batches A–C	Sampled Fraction	Yield (%) Batches A1, A2	Yield (%) Batches B1, B2
Unprocessed raw maize ^a^	1	100	1	100	100
Rejected fraction from separator ^b^		-	2	3.0	2.0
Rejected fraction from separator ^c^		-	3	0.9	0.9
Rejected fraction from aspirator ^d^		-	4	0.5	0.5
Rejected fraction from concentrator ^e^		-	5	1.5	0.5
Rejected fraction from optical sorter ^f^	2	5.0	6	0.5	0.1
Cleaned maize	3	95.0	7	93.6	96.0

^a^ in study 1 the incoming maize was slightly pre-cleaned by mechanical sorting; ^b^ small, broken and fine materials; ^c^ coarse and fine materials; ^d^ dust and husks particles; ^e^ low density maize kernels; ^f^ coloured/discoloured and defective maize kernels.

**Table 2:** Effect of industrial-scale sorting by the SORTEX A5 optical sorter on deoxynivalenol (DON), zearalenone (ZEA) and total fumonisins (FBs) content in sampled fractions.

Batch	Sampled Fraction ^1^	DON (µg/kg)	DON Reduction (%)	ZEA (µg/kg)	ZEA Reduction (%)	FBs ^2^ (µg/kg)	FBs ^2^ Reduction (%)
A	1	11,130	63	2690	78	5680	27
	2	108,540		40,310		22,850	
	3	4100		580		4150	
B	1	17,400	67	4460	87	6540	28
	2	168,250		18,700		22,320	
	3	5790		590		4690	
C	1	3200	44	660	67	2520	27
	2	27,620		10,060		7860	
	3	1780		220		1830	

^1^ Fraction 1 input optical sorter (slightly pre-cleaned by mechanical sorting); fraction 2: rejected fraction from optical sorter (coloured and discoloured and defective maize kernels); fraction 3: cleaned maize (end product); ^2^ sum of fumonisin B1 and fumonisin B2.

**Table 3:** Effect of industrial-scale cleaning line (separator-aspirator-concentrator-optical sorter) on deoxynivalenol (DON), zearalenone (ZEA) and total fumonisins (FBs) content in sampled maize fractions from batch A (French maize) and batch B (Spanish maize). Two replicates per batch were carried out, i.e., A1, A2, B1 and B2.

Batch	Sampled Fraction ^1^	DON (µg/kg)	DON Reduction (%)	ZEA (µg/kg)	ZEA Reduction (%)	FBs ^2^ (µg/kg)	FBs ^2^ Reduction (%)
A1	1	250	36	50	80	1705	54
	2	940		160		7640	
	3	1260		265		14,180	
	4	1350		270		8100	
	5	2600		540		31,760	
	6	5550		90		3640	
	7	160		10		780	
A2	1	220	52	40	75	1765	34
	2	680		170		6830	
	3	1490		230		19,720	
	4	1300		130		17,305	
	5	1560		350		28,890	
	6	2550		490		5620	
	7	105		10		1160	
B1	1	350	43	55	82	1740	67
	2	1080		115		4870	
	3	4240		550		48,250	
	4	10,680		1405		35,575	
	5	5760		760		52,530	
	6	12,280		750		8240	
	7	200		10		580	
B2	1	330	48	50	80	1735	50
	2	1030		55		4740	
	3	4970		560		44,980	
	4	9900		180		40,670	
	5	5820		250		39,990	
	6	11,230		745		7400	
	7	170		10		860	

^1^ Fraction 1: unprocessed maize (incoming material); Fraction 2: rejected fraction from separator (small, broken and fine material); Fraction 3: rejected fraction from separator (coarse and fine material); Fraction 4: rejected fractions from aspirator (dust and husks particles); Fraction 5: rejected fraction from concentrator (low density kernels); Fraction 6: rejected fraction from optical sorter (coloured and discoloured and defective maize kernels); Fraction 7: cleaned maize (end product); ^2^ sum of fumonisin B1 and fumonisin B2.

**Table 4:** Mass balance (%) of deoxynivalenol (DON), zearalenone (ZEA) and total fumonisins (FBs) in studies 1 and 2.

Study	Batch	DON (%)	ZEA (%)	FBs ^1^ (%)
1	A	84	95	90
	B	80	87	85
	C	96	108	85
2	A1	105	53	95
	A2	79	62	114
	B1	99	43	88
	B2	96	37	100

^1^ sum of FB_1_ and FB_2_.

**Table 5:** Number of incremental samples of sampled fractions and weight of the aggregate samples, according to the Commission Regulation (EU) No 401/2006.

Study	Sampled Fraction	Number of Incremental Samples	Aggregate Sample Weight (kg)
1 ^1^	1, 3	100	10–14
	2	10	1–2
2 ^2^	1, 7	60	6–10
	2	10	1–2
	3, 4, 5, 6	5	1–2

^1^ Batches of 25 tons. Fractions: 1, unprocessed maize; 2, foreign bodies and kernels with visual defects from sorter; 3, cleaned maize. ^2^ Batches of 17 tons. Fractions: 1, unprocessed maize: 2, small, broken and fine material from separator; 3, coarse and fine material from separator; 4, dust and husks particles from aspirator; 5, lighter maize fractions from concentrator; 6, maize kernels with visual signs of contamination; 7, cleaned maize.

## Data Availability

Data sharing not applicable.
